# 1461. The Impact of Universal Varicella Vaccination on Herpes Zoster Incidence in the United States

**DOI:** 10.1093/ofid/ofac492.1288

**Published:** 2022-12-15

**Authors:** Jessica W Leung, Kathleen L Dooling, Mona Marin, Tara C Anderson, Rafael Harpaz

**Affiliations:** Centers for Disease Control and Prevention, Atlanta, Georgia; Centers for Disease Control and Prevention, Atlanta, Georgia; Centers for Disease Control and Prevention, Atlanta, Georgia; Centers for Disease Control and Prevention, Atlanta, Georgia; Harpaz-Herman Consultants, Atlanta, Georgia

## Abstract

**Background:**

The varicella vaccination program was implemented in the United States in 1995 and has resulted in large reductions in varicella disease burden. At the start of the varicella vaccination program, impacts on herpes zoster (HZ) epidemiology were unknown. We used a large claims database to examine national HZ incidence during 1998–2019 in cohorts of persons born before and after varicella vaccine introduction.

**Methods:**

Medical claims data were obtained from 1998–2019 IBM® MarketScan® Research Databases. We identified HZ based on first outpatient service with an HZ ICD-9/10 code (053.xx/B02.xx) and calculated age-specific incidence for persons aged ≥30 years (all born pre-vaccine) and persons aged 1–29 years (includes persons born post-vaccine).

**Results:**

Among persons aged ≥30 years, HZ incidence increased with age and calendar time (Fig. 1); incidence in the two oldest age groups (60–69 and ≥70 years) started to decelerate in 2007. Among persons aged 1–29 years, HZ incidence increased early in the study period for the oldest age groups (born pre-vaccine, i.e., born after 1990), but later declined once each age group was comprised of persons born in the post-vaccine period (children and adolescents) (Fig 2); the peak incidence occurred in progressively-earlier calendar years among progressively younger age groups.

**Figure:**
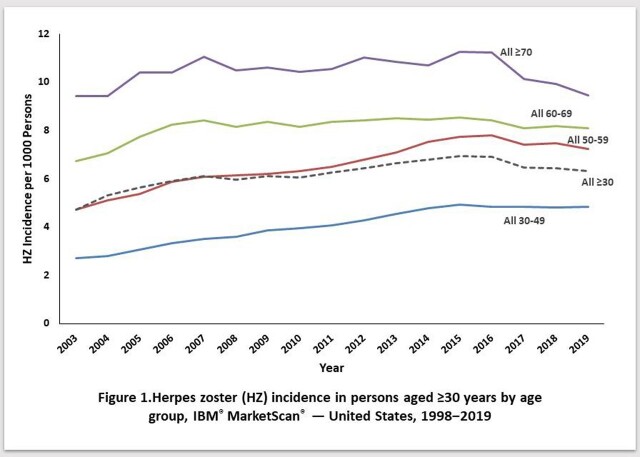

**Figure:**
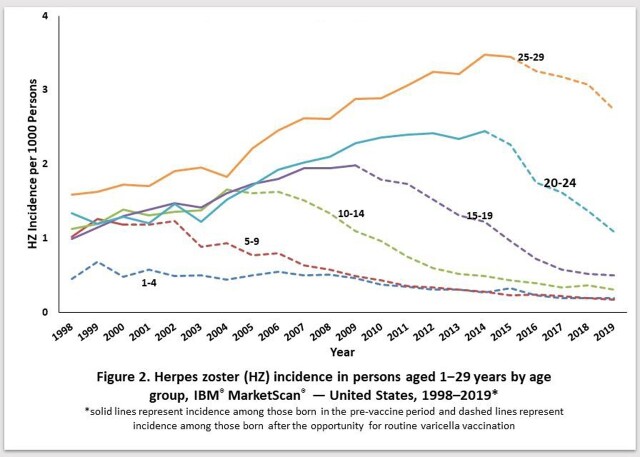

**Conclusion:**

HZ incidence in persons aged ≥30 years increased early in the study period for all age groups followed by declines starting with the oldest cohorts. The U.S. data do not support previous modelled predictions that reduced VZV exposure would result in increased HZ incidence among adults. The patterns of decline in HZ incidence in persons aged 1–29 years can likely be explained by the success of the U.S. varicella vaccination program and increasingly uncommon latent infection with wild type varicella-zoster virus. These data suggest that continued declines in age-specific HZ incidence are likely as varicella vaccinated cohorts age. The reduction of HZ resulting from the varicella vaccination program, should ultimately extend to the entire U.S. population over time.

**Disclosures:**

**All Authors**: No reported disclosures.

